# Development and Application of a Prioritization Tool for Animal Health Surveillance Activities in Ireland

**DOI:** 10.3389/fvets.2020.596867

**Published:** 2020-12-23

**Authors:** AnneMarie Clarke, Simon J. More, James W. Maher, Andrew W. Byrne, Michael Horan, Damien Barrett

**Affiliations:** ^1^One Health One Welfare Scientific Support Unit, Department of Agriculture, Food and the Marine, Agriculture House, Dublin, Ireland; ^2^Centre of Veterinary Epidemiology and Risk Analysis, School of Veterinary Medicine, University College Dublin, Dublin, Ireland

**Keywords:** disease prioritization, surveillance, animal health, endemic diseases, exotic diseases

## Abstract

Decisions around animal health management by stakeholders are often subject to resource limitation, therefore prioritization processes are required to evaluate whether effort is attributed appropriately. The objectives of this study were to develop and apply a surveillance prioritization process for animal health surveillance activities in Ireland. An exploratory sequential mixed research methods design was utilized. A prioritization tool was developed for surveillance activities and implemented over two phases. During the first phase, a survey was conducted which asked stakeholders to prioritize diseases/conditions by importance for Irish agriculture. In the second phase, experts identified the most important surveillance objectives, and allocated resources to the activities that they considered would best meet the surveillance objectives, for each disease/condition. This study developed a process and an accompanying user-friendly practical tool for animal disease surveillance prioritization which could be utilized by other competent authorities/governments. Antimicrobial resistance and bovine tuberculosis were ranked top of the endemic diseases/conditions in the Irish context, while African swine fever and foot and mouth disease were ranked top of the exotic diseases/conditions by the stakeholders. The study showed that for most of the diseases/conditions examined in the prioritization exercise, the respondents indicated a preference for a combination of active and passive surveillance activities. Future extensions of the tool could include prioritization on a per species basis.

## Introduction

International experience has found that prioritization of expenditure on animal health is challenging but it is an important activity to ensure that resources are appropriately attributed (1–3). Furthermore, such prioritization efforts can contribute to multi-stakeholder engagement and network building. These are particularly important for animal health in countries where agriculture makes a large social and economic contribution, for example, in Ireland and also in countries that are particularly limited in terms of resources. Agri-food is Ireland's most important indigenous industry, playing a vital role in the national economy. In 2018, agriculture accounted for €7.9 billion of gross output and 81.2% of this emanated from the livestock industry. In December 2019 there were 6.4 million cattle (dairy and beef), 3.8 million sheep and 1.6 million pigs in Ireland (4). In the last farm structure survey carried out in December 2016, it was recorded that there were over 11 million poultry in Ireland (5). Ireland relies heavily on its exports and many of its products are produced well in excess of self-sufficiency, particularly in the livestock industry (6).

Animal health surveillance plays an essential role in supporting the livestock industry in Ireland, while also contributing to the protection of public health and environmental well-being. Animal health surveillance has been defined as “the systemic collection, collation, analysis, interpretation and timely dissemination of animal and welfare data from defined populations” (7). Helping to maintain and enhance export markets is one of the main roles of animal health surveillance and prioritizing surveillance activities is critical to maximize the use of available resources (8). Resources (both financial and human) are limited; therefore, it is necessary to invest in surveillance activities that are of priority and de-emphasize those that no longer add value (9).

Brookes et al. (10) reviewed the progression of disease prioritization methodologies, which included *ad hoc* procedures and decision science methods (such as multi-criteria decision analysis and probabilistic analysis). The RiskSur project referenced the Swedish model as an example of best practice, however it suggested that “even with the best prioritization models, the outcome should never be seen as the absolute truth but rather as an informed input to tactical and strategic decision- making.” It was noted that priorities change overtime and the prioritization process needs to be repeated at regular intervals (10, 11).

In Ireland there is no formal prioritization process for animal health surveillance activities and to date it has been mainly guided by legislative requirements and internal decisions by the Ministry responsible for animal health. This was recognized in a key recommendation of the Animal Health Surveillance Strategy for Ireland 2016–2021 (9), which stated that “*DAFM should develop a prioritization process for animal health surveillance activities*.” Therefore, the objective of this study was to develop and apply a prioritization tool for animal health surveillance activities in Ireland.

## Materials and Methods

### Tool Development and Piloting

**Figure 1:**
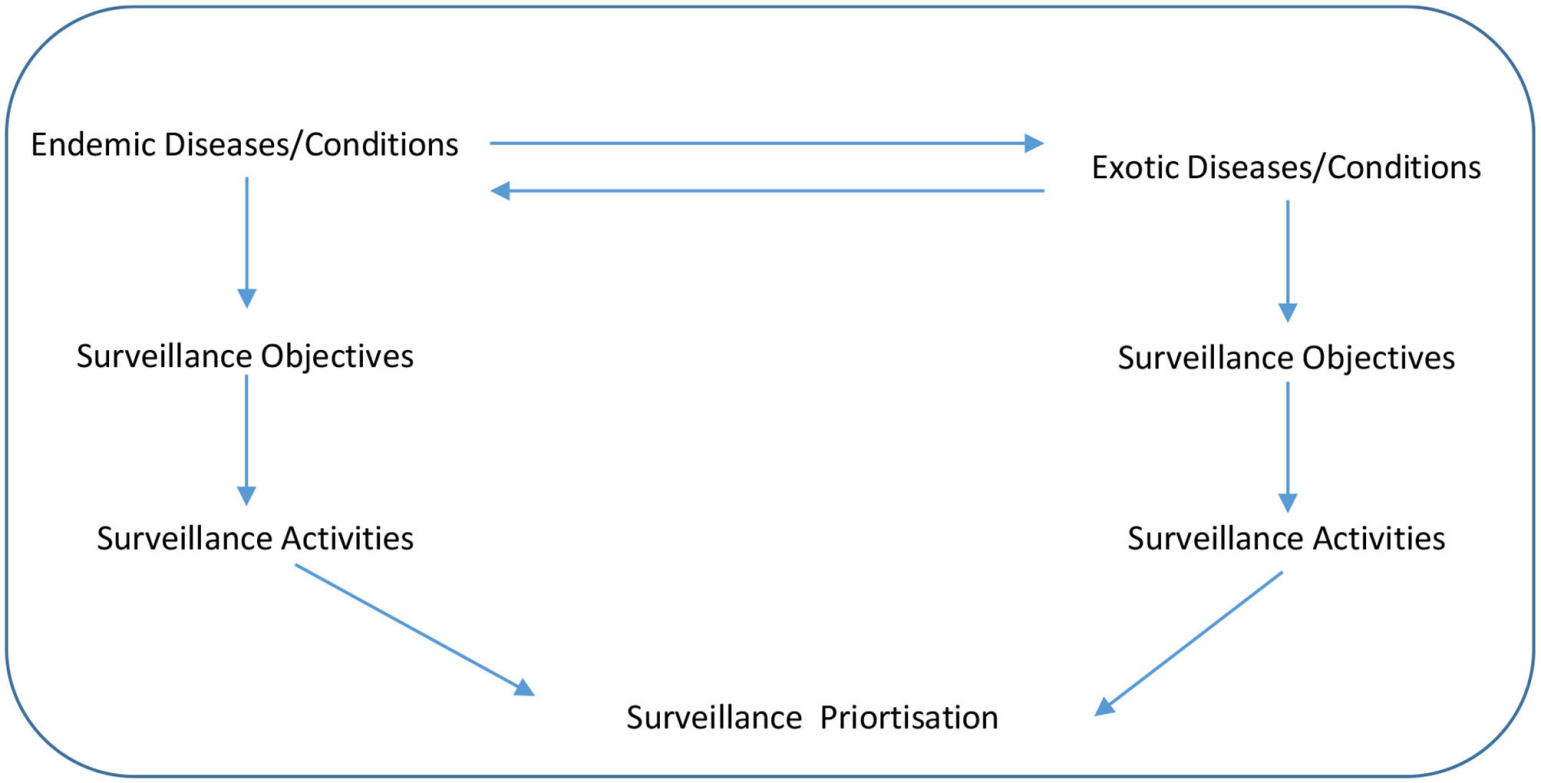
Conceptual framework for the animal health surveillance priortization.

An exploratory sequential mixed research methods design was applied over two workshops during the piloting and development step. The tool (in which users could complete the prioritization exercise) was drafted by the lead author and then further developed during the first workshop with the co-authors. At this first workshop, there was a structured, agenda-led discussion around the surveillance prioritization process, including consideration of the surveillance objectives, specific diseases and surveillance activities. A conceptual framework for animal health surveillance prioritization that the tool would address is outlined in Figure 1. The direction of the arrows between the endemic and exotic diseases show that a disease can change from being exotic to endemic if it is allowed to spread in a county, while a disease can also change over time from being endemic to exotic if eradication programmes are successful. The downward arrows shows the flow of what must be considered in surveillance prioritization, firstly the disease itself, secondly, what are the surveillance objectives for this disease and thirdly, what surveillance activities best meet the surveillance objectives for the disease. It was agreed that the tool should be simple (using pre-existing enabling technology such as spreadsheets and supported by online surveys), timely and transparent (in terms of scoring and feedback to contributors) with an ability to prioritize surveillance activities for a range of diseases and conditions.

A second workshop was conducted. The aims of this workshop were to pilot test the developed tool and agree on any modifications that would help improve its effectiveness. The workshop consisted of seven experts working in the animal health surveillance division and One Health One Welfare scientific support unit within the Irish Department of Agriculture, Food and the Marine (DAFM). These experts (requested to participate based on convenience sampling) had veterinary, epidemiology and social science backgrounds, and those with veterinary expertise had experience in working on surveillance programmes. The tool was emailed to them in the EXCEL spreadsheet format (Microsoft Excel 2013 Microsoft Corporation, Redmond, WA, USA) a week in advance of the workshop and so that they had completed the prioritization exercise using the tool before discussing it at the workshop. The prioritization exercise consisted of the users of the tool assigning values to the surveillance objectives and surveillance activities for each of the disease listed. The discussions at the workshop resulted in several recommendations which were incorporated into the tool. Specifically, issues around partitioning/grouping diseases and approaches to ranking were evaluated. The three key modifications were:

1. It was originally planned to score the surveillance objectives on two criteria, cost and effectiveness, however rather than scoring the surveillance objectives (case finding and prevalence estimation for endemic diseases; and early detection and proof of freedom for exotic diseases) on these criteria, the objectives should be scored using a priority weight on importance. Each disease will be examined for two surveillance objectives and the total weight should add to 10. The experts using the tool can apply respective weights to each objective.
2. Under surveillance activities, it was agreed that experts should allocate resources to the activities that best meet the surveillance objectives and in doing so they may refer to the Centers for Disease Control and Prevention guidelines (12) when considering the activities and the allocation of resources to these activities during the prioritization exercise. This modification meant that the previous criteria of cost and effectiveness were removed from the tool altogether. The justification for this was that only a limited number of experts would actually know the costs associated with the surveillance activities and examining the effectiveness of the surveillance activities is more appropriate for an evaluation of activities rather than the prioritization of activities.
3. Given that the two criteria (cost and effectiveness) were removed from the tool, it was necessary to identify a method in which the experts could allocate resources the surveillance activities. Ranking of surveillance activities would be undertaken using a proportional piling technique (13, 14). This technique offers a method of assigning a relative priority or value to parameters. There is a limited amount of resources (financial and human) available for surveillance activities therefore one must allocate the resources to the activities within these limits. Experts would be asked to allocate the resources across the activities by assigning “chips” to the relevant activities for each disease. Using the proportional piling technique, 100 chips were available for each disease and these should be considered as the total amount of resources (financial and human) currently available for each disease.

### Implementation

#### The First Phase

The first implementation phase was framed around the identification of the most important diseases that the Irish government should prioritize for national animal health surveillance activities. A self-completion survey using the *SurveyMonkey* software package (SurveyMonkey, San Mateo, California) was created and issued to sixty-two stakeholders. The stakeholders consisted of five groups, including farmer representatives (for example members of Irish Farmers Association), state agency staff (members of Teagasc, Bord Bia, for example), farm service providers (Irish Cattle Breeding Federation), private veterinarians (mixed practitioners) and relevant senior government veterinary staff. The email addresses of a stakeholder group, which provided guidance to the animal health surveillance division within DAFM was made available, as DAFM regularly consults with them on surveillance matters. These addresses included stakeholders for four of the groups above and the email addresses of the relevant senior government veterinary staff were readily available to the authors, all of which were invited to participate. There were six questions to be answered in this phase, which are detailed in the Supplementary Material A. An email with a link to the survey was issued to these stakeholders. Consent to participate on a confidential basis was obtained from the survey respondents in the first question of the survey. Two weeks were allowed for the completion of the survey and a reminder email was issued 1 week in advance of the deadline. The outcome of this phase was a ranked list of the important endemic (Table 1) and exotic (**Table 3**) disease/conditions which were then inputted into the prioritization tool.

**Table 1:** Ranking of endemic diseases/conditions in Ireland by stakeholders in the animal health sector.

**Ranking**	**Endemic Disease/condition**	**Frequency**	**Weighted Average^1^**	**Importance^2^**
1	Antimicrobial Resistance (AMR)	31	8.23	255
2	Bovine TB	31	7.58	235
3	Respiratory diseases	25	6.8	170
4	Johne's disease	26	4.62	120
5	Salmonellosis	23	5.22	120
6	Parasitism	23	5.13	118
7	Liver fluke	21	5.1	107
8	Neonatal enteritis	17	6.12	104
9	Bovine Viral Diarrhea (BVD)	22	4.68	103
10	Infectious Bovine Rhinotracheitis (IBR)	16	5.06	81
11	*E. coli* 157	19	3.74	71
12	Bovine Spongiform Encephalopathy (BSE)	12	5.75	69
13	Campylobacteriosis	12	5.67	68
14	Porcine Reproductive and Respiratory Syndrome (PRRS)	11	4.45	49
15	Clostridial diseases e.g., Botulism or Blackleg	10	3.7	37
16	Digital dermatitis	10	3	30
17	Scrapie	4	4.5	18
18	Schmallenburg Virus	4	2.75	11
19	Bovine genital campylobacteriosis	2	5	10
20	Besnoitia	2	3.5	7

1 *Weighted average was calculated using reverse scoring, for example if a disease was ranked as 1st place then the weight was 10. If a disease was ranked in 10th place the weight was 1. The average of these weights were used in the calculation of importance*.

2 *Calculation of importance, this is a product of frequency of response multiplied by weighted average*.

#### The Second Phase

The second implementation phase sought to allow experts in the field of animal health surveillance to complete the prioritization exercise for the twenty diseases identified during phase one. The ten most important endemic diseases and ten most important exotic diseases were inputted into the tool. A guidance document was prepared on how to complete the prioritization exercise using the developed tool. A copy of this document is presented in the Supplementary Material B. The prioritization tool and accompanying guidance document were emailed to twelve experts requesting them to complete the exercise in the excel spreadsheet. The experts included academics (a professor of Veterinary Epidemiology and Risk Analysis) and senior official veterinarians with responsibility for animal health and welfare policies located within Republic of Ireland and Northern Ireland. All were knowledgeable about animal health surveillance activities in Ireland and consent to participate on a confidential basis was obtained beforehand.

### Data Analysis

The ranking of the top ten diseases/conditions for both endemic and exotic groups were provided. Analysis of these data involved the comparison of the ranking of diseases/conditions between government and non-government respondents. For phase two, data were collated and sorted, and the mean, minimum and maximum values were calculated for the surveillance objectives and activities for each disease (ten endemic diseases and ten exotic diseases).

## Results

### Tool Development and Piloting

The specific generic tool structure was developed within a spreadsheet application, an example of which is presented in the Supplementary Material C. The development and piloting step identified several challenges for the tool, including grouping and ranking activities. Feedback during the piloting identified the following grouping and surveillance ranking approaches, which were incorporated into the tool. The tool partitions two categories of diseases (or conditions), on two separate sheets within the excel spreadsheet namely endemic and exotic, given their differing surveillance objectives (endemic: prevalence estimation and case finding; exotic: early detection and proof of freedom). For each category, the prioritization tool comprised two parts. Firstly, it involved examining which surveillance objective is of a higher priority for each disease. Secondly, it examined which surveillance activities are best suited to meet the surveillance objectives for each disease within the constraints of the resources available for disease surveillance. Detailed guidelines on how to complete the exercise are provided within the tool on the spreadsheet (Supplementary Material C).

### First Implementation Phase

Thirty-four respondents completed the survey using SurveyMonkey, giving a response rate of 54.8%. The five categories of stakeholders included farmer representatives 3%, state agency staff 32%, farm service providers 6%, private veterinarians 6%, and government staff 53%. There was almost a 50-50 split between the number of government respondents and commercial stakeholders (non-government).

The frequency, weighted average and importance, and the resulting ranking of the endemic diseases/conditions are presented in Table 1. Antimicrobial resistance (AMR), bovine TB and respiratory diseases were considered as the three most important endemic diseases/conditions, by the respondents. AMR and bovine TB were ranked the same number of times (frequency = 31), however AMR was ranked higher than bovine TB during this ranking by the respondents resulting in a calculated importance of 255 vs. 235, respectively. There was a good relationship between the government and the non-government respondents. As presented in Table 2, shaded diseases/conditions are those that both cohorts ranked in the top ten of the endemic diseases. Antimicrobial resistance, bovine TB and respiratory diseases were considered the top three for both cohorts although the sequence did differ for the first and second ranking. Bovine TB was ranked first by government respondents while AMR was given first place by the non-government respondents.

**Table 2:** Comparison of government vs. non-government respondents on the ranking of endemic disease/conditions.

**Ranking**	**Endemic Disease/Condition**	
	**Government only**	**Non-government**
1	Bovine TB	Antimicrobial Resistance (AMR)
2	Antimicrobial Resistance (AMR)	Bovine TB
3	Respiratory diseases	Respiratory diseases
4	Liver fluke	Salmonellosis
5	Bovine Viral Diarrhea (BVD)	Johne's disease
6	Parasitism	Neonatal enteritis
7	Salmonellosis	Parasitism
8	Johne's disease	Liver fluke
9	Infectious Bovine Rhinotracheitis (IBR)	Bovine Viral Diarrhea (BVD)
10	Bovine Spongiform Encephalopathy (BSE)	Infectious Bovine Rhinotracheitis (IBR)
11	*E. coli* 157	*E. coli* 157
12	Campylobacteriosis	Clostridial diseases e.g., Botulism or Blackleg
13	Neonatal enteritis	Campylobacteriosis
14	Porcine Reproductive & Respiratory Syndrome (PRRS)	Porcine Reproductive & Respiratory Syndrome (PRRS)
15	Digital dermatitis	Bovine Spongiform Encephalopathy (BSE)
16	Scrapie	Digital dermatitis
17	Clostridial diseases e.g., Botulism or Blackleg	Bovine genital campylobacteriosis
18	Schmallenburg Virus	Schmallenburg Virus
19	Besnoitia	Besnoitia
20	Bovine genital campylobacteriosis	Scrapie

*The shaded diseases are those diseases that were selected by both government and non-government respondents which were ranked in the top 10 most important endemic diseases/condition*.

The results from the phase one survey for exotic diseases are presented in Table 3. African swine fever (ASF), foot and mouth disease (FMD), and the bluetongue virus (BTV) were considered as the three most important exotic diseases/conditions, while bovine brucellosis was ranked fourth with the same level of importance as BTV. The relationship between the government respondents and the non-government respondents was not as strong as that with the endemic diseases. In Table 4, the shaded diseases/conditions are those that both cohorts ranked in the top ten of the exotic diseases. Both cohorts ranked the same seven diseases in their top ten; however the order of the ranking differed for all the diseases. ASF and FMD were placed in the top three by both cohorts.

**Table 3:** Results of the ranking of exotic diseases/conditions in Ireland by stakeholders in the animal health sector.

**Ranking**	**Exotic Disease/Condition**	**Frequency**	**Weighted Average^2^**	**Importance^3^**
1	African Swine Fever (ASF)	32	7.97	255
2	Foot and Mouth Disease (FMD)	32	7.50	240
3	Bluetongue Virus (BTV)	26	6.27	163
4	Bovine brucellosis	25	6.52	163
5	Rabies	27	5.59	151
6	Classical Swine Fever (CSF)	27	5.37	145
7	Avian Influenza	24	5.92	142
8	Dioxin contamination of pork meat	13	5.38	70
9	Equine Infectious Anemia (EIA)	17	4.06	69
10	Equine Viral Arteritis (EVA)	16	4.13	66
11	Disease X*^1^	13	5.08	66
12	African Horse Sickness	16	4.06	65
13	Enzootic Bovine Leukosis (EBL)	19	3.16	60
14	Anthrax	11	5.36	59
15	Fowl Typhoid (*Salmonella gallinarum*)	10	4.70	47
16	Aujeszky's Disease	12	3.33	40
17	Rift Valley Fever	8	3.00	24
18	Brucella melitensis	5	4.40	22
19	Caprine arthritis encephalitis	5	3.20	16
20	Equine piroplasmosis	2	3.50	7

1 *Disease X is a previously unrecognized disease, which could first emerge in Ireland*.

2 *Weighted average was calculated using reverse scoring, for example if a disease was ranked as 1st place then the weight was 10. If a disease was ranked in 10th place the weight was 1. The average of these weights were used in the calculation of importance*.

3 *Calculation of importance, this is a product of frequency of response multiplied by weighted average*.

**Table 4:** Comparison of government vs. non-government respondents on the ranking of exotic disease/conditions.

**Ranking**	**Exotic Disease/Condition**	
	**DAFM only**	**Non-DAFM**
1	Foot and Mouth Disease (FMD)	African Swine Fever (ASF)
2	African Swine Fever (ASF)	Bovine brucellosis
3	Bluetongue Virus (BTV)	Foot and Mouth Disease (FMD)
4	Avian Influenza	Classical Swine Fever (CSF)
5	Rabies	Rabies
6	Classical Swine Fever (CSF)	Bluetongue Virus (BTV)
7	Bovine brucellosis	Anthrax
8	Equine Viral Arteritis (EVA)	Avian Influenza
9	Equine Infectious Anemia (EIA)	African Horse Sickness
10	Disease X*^1^	Dioxin contamination of pork meat
11	Dioxin contamination of pork meat	Enzootic Bovine Leukosis (EBL)
12	Enzootic Bovine Leukosis (EBL)	Fowl Typhoid (*Salmonella gallinarum*)
13	African Horse Sickness	Disease X*
14	Rift Valley Fever	Aujeszky's Disease
15	Fowl Typhoid (*Salmonella gallinarum*)	Equine Infectious Anemia (EIA)
16	Aujeszky's Disease	Equine Viral Arteritis (EVA)
17	*Brucella melitensis*	*Brucella melitensis*
18	Caprine arthritis encephalitis	Equine piroplasmosis
19	Anthrax	Caprine arthritis encephalitis
20	Equine piroplasmosis	Rift Valley Fever

1 *Disease X is defined as a previously unrecognized disease, which could first emerge in Ireland*.

*The shaded diseases are those diseases that were selected by both government and non-government respondents which were ranked in the top 10 most important endemic diseases/condition*.

### Second Implementation Phase

Ten experts completed the prioritization exercise for the endemic diseases, while eight completed it for the exotic diseases. The mean scores (out of 10) for the surveillance objectives are presented in Table 5 for each disease. Prevalence estimation was a greater priority for diseases/conditions such as AMR, neonatal enteritis, parasitism including liver fluke, and respiratory diseases. In contrast, the experts scored “case finding” as the more important objective for BSE, TB and BVD. For the exotic diseases, experts prioritized the objective “early detection” over “proof of freedom” for ASF, BTV, CSF, disease X, equine infectious anemia (EIA), equine viral arteritis (EVA), FMD and rabies, allocating a mean score of over 6.5 for each disease.

**Table 5:** Mean score in the prioritization of surveillance objectives for endemic and exotic diseases/conditions by experts completing the prioritization exercise.

**Diseases/conditions**	**Surveillance objectives**		**Surveillance objectives**	
	**Case finding (for eradication)**	**Prevalence estimation (for monitoring)**	**Early detection**	**Proof of freedom**
**ENDEMIC**				
Antimicrobial Resistance (AMR)	2.6	7.4	N/A	N/A
Bovine Spongiform Encephalopathy (BSE)	7	3	N/A	N/A
Bovine TB	7.9	2.1	N/A	N/A
Bovine Viral Diarrhea (BVD)	7.6	2.4	N/A	N/A
Equine herpesvirus infection	4.6	5.4	N/A	N/A
Infectious Bovine Rhinotracheitis (IBR)	4.9	5.1	N/A	N/A
Johne's disease	5.7	4.3	N/A	N/A
Neonatal enteritis	2.2	7.8	N/A	N/A
Parasitism including liverfluke	2.4	7.6	N/A	N/A
Respiratory diseases	2.3	7.7	N/A	N/A
**EXOTIC**				
African Swine Fever (ASF)	N/A	N/A	8.2	1.8
Avian Influenza	N/A	N/A	6.6	3.4
Bluetongue Virus (BTV)	N/A	N/A	6.7	3.3
Bovine brucellosis	N/A	N/A	6.2	3.8
Classical Swine Fever (CSF)	N/A	N/A	7.4	2.6
Disease X *	N/A	N/A	9.3	0.8
Equine Infectious Anemia (EIA)	N/A	N/A	6.8	3.2
Equine Viral Arteritis (EVA)	N/A	N/A	6.6	3.4
Foot and Mouth Disease (FMD)	N/A	N/A	8.7	1.3
Rabies	N/A	N/A	8.7	1.3

*Disease X is defined as a previously unrecognized disease which could first emerge in Ireland*.

*The values presented represent the mean score (ranging 1 to 10) which experts assigned to each objective for each disease. The max score across the two objectives for each disease was 10*.

The allocation of resources between active and passive surveillance activities for endemic and exotic diseases/conditions were summarized in Table 6. The figures in the table represent the mean number of chips allocated to the active and passive activities for each disease/condition by the experts. A greater number of resources were allocated to the active surveillance activities compared to the passive activities across the endemic diseases. AMR was the only condition that had equal resources allocated between both types of surveillance groups. For exotic diseases, experts allocated higher resources for active surveillance activities for avian influenza, BTV, bovine brucellosis and CSF. Equal amounts of resources were allocated to passive and active surveillance activities for ASF and rabies. Higher resources were allocated to passive surveillance for the remainder of the diseases, namely disease X, equine infectious anemia, equine viral arteritis and FMD. The experts allocated 70% of the resources to active surveillance activities for BTV. For all diseases/conditions, the results suggested that a combination of active and passive surveillance activities were required. A detailed breakdown of the allocation of recourses to individual surveillance activities are provided in the Supplementary Material D.

**Table 6:** Allocation of resources to active vs. passive activities for endemic and exotic diseases/condition by experts completing the prioritization exercise.

**Diseases/conditions**	**Surveillance activities**	
	**Active**	**Passive**
**ENDEMIC**		
Antimicrobial Resistance (AMR)	50	50
Bovine Spongiform Encephalopathy (BSE)	54	46
Bovine TB	76	24
Bovine Viral Diarrhea (BVD)	76	24
Equine herpesvirus infection	35	65
Infectious Bovine Rhinotracheitis (IBR)	65	35
Johne's disease	67	33
Neonatal enteritis	18	82
Parasitism including liverfluke	62	38
Respiratory diseases	41	59
**EXOTIC**		
African Swine Fever (ASF)	50	50
Avian Influenza	56	44
Bluetongue Virus (BTV)	70	30
Bovine brucellosis	55	45
Classical Swine Fever (CSF)	60	40
Disease X *	29	71
Equine Infectious Anemia (EIA)	41	59
Equine Viral Arteritis (EVA)	36	64
Foot and Mouth Disease (FMD)	41	59
Rabies	50	50

*Disease X is defined as previously unrecognized disease which could first emerge in Ireland*.

*The figures in the table represent the mean number of chips allocated to the active and passive activities for each disease/condition*.

## Discussion

This study marked the development of a novel animal health surveillance activities prioritization process in Ireland, which could be applied in other countries. The application of the tool to animal health surveillance priorities in Ireland found that AMR and bovine TB emerged as the two most important endemic diseases from the stakeholder consultation. This was unsurprising and consistent with the literature, for example, Prestinaci et al. (15) and Núñez-Núñez et al. (16) reported that AMR is one of the greatest global public health concerns in recent years. TB is a regulated disease with ongoing surveillance programmes in place (17–19) for several years with large amounts of resources being allocated to these programmes (20). ASF and FMD emerged as the two most important exotic diseases from the stakeholder consultation, which again was not surprising. In recent years the threat of ASF has increased due to its spread across Europe (21, 22), with the potential to devastate the Irish pig industry (23). Similarly, the high ranking of FMD suggests the importance of having a robust surveillance programme in place for that disease. Recent experience demonstrates the significant negative economic consequences of FMD outbreaks (24).

Experts allocated resources to a combination of both active and passive surveillance activities for many of the diseases, while often the proportion of resources were greater for the active surveillance activities in particular for the endemic diseases. This may be related to a perceived higher cost of active surveillance but more likely reflecting the higher importance of active surveillance in terms of efficiency for the stated goals. The combination of both active and passive surveillance activities was often consistent with the current practices already in place for the surveillance of these diseases. For example, the case finding objective was the priority objective for TB, BSE and BVD. Currently there is a legislative basis (national and EU) for the eradication of TB and BSE, and there is a national eradication programme for BVD in Ireland (25). The higher amounts of resources put into active surveillance activities for TB may be explained by the requirement for this under regulations around the surveillance of TB. Under legislation for TB, there is a requirement to test all cattle each year in Ireland. Similarly for BVD, the higher amounts of resources in active surveillance can be attributed to the national eradication programme which currently requires an individual animal test status for an animal to be traded. For BSE, there was a relatively even split of resources assigned to passive and active surveillance programmes. This probably reflects the relative importance assigned to the investigation of clinical suspects which would be considered a higher risk category for investigation. The OIE is currently considering proposals to move toward a more passive based surveillance system for BSE, in the context of the disease being at a historically low incidence (Barrett, personnel communication). The experts allocated 70% of the resources to active surveillance activities for BTV. The greater allocation of resources to active surveillance activities may be linked to the greater potential impact on the trade of live animals and animal products such as semen and embryos. The economic losses associated with this disease can be substantial at farm level through reduced milk yield and animal performance and abortions (26), However losses incurred due to the likely impact on trade would be considerably higher, given Ireland's reliance on export markets.

Application of the tool has led to evidence-based recommendations which will guide future animal health surveillance prioritization. For example, during phase one of implementation, stakeholders were provided with an opportunity to input into the strategic direction of animal health surveillance activities when government is selecting the diseases to be included in national surveillance programmes. For phase two, a more in depth understanding of surveillance activities was required and, for this reason, only experts with experience of such activities were invited to participate. Results from phase two of implementation suggest that some experts had a greater knowledge of their own areas of responsibility and were not familiar with areas outside their own remit. For example, two of the ten experts did not complete all the prioritization exercise. Furthermore, this would suggest that more consideration in the selection of such experts and their knowledge of particular diseases may be needed. This could be addressed by choosing experts with in-depth knowledge of specific diseases. Indeed, completing the prioritization tool on a per species basis rather than a broad sweep of diseases/conditions may be more appropriate. However, the disadvantage to that approach may be that there could be an over reliance on single experts rather than considering the opinion of a number of experts together, and confining it to such subject matter experts could make the group more likely to stick with the status quo and not to be as open to change.

While the application of the prioritization process and accompanying developed tool has addressed one of the recommendations of the Animal Health Surveillance Strategy for Ireland 2016–2021, this process might also assist Ireland and other EU Member States in addressing their obligations under the forthcoming Animal Health Law in 2021. It must be noted that the specific outputs for specific diseases may change over time. However, this study has established a process and it can be easily repeated. Indeed, Lomas et al. (27) stated that “the process is more important than the science” when describing prioritization efforts. Over time, the objectives of the surveillance programmes may change, therefore it was necessary that these were considered prior to considering the surveillance activities themselves. For example, in the case of bovine brucellosis in Ireland, the surveillance objectives changed over time with the shift from control of an endemic disease (with a surveillance objective of case-finding) through to eradication (where the objective is proof of freedom). This example also demonstrates the need for the inclusion of both endemic and exotic diseases, and related surveillance objectives and activities, as part of the framework for surveillance prioritization. It is anticipated that this prioritization process will be repeated in the future, allowing trends over time to be evaluated.

## Conclusions

This study has led to the development of the prioritization process and accompanying tool for animal health surveillance activities. This process can be repeated and aligned at regular intervals with the epidemiological situation for a disease (i.e., as the epidemiological situation evolves, the surveillance activities will also have to evolve). The exercise of seeking a range of stakeholder opinions on disease prioritization (phase one) was considered inclusive. This phase ensures decisions made by government and the department that is responsible for animal disease surveillance programmes can be aligned with the views of experts across veterinary divisions in government and the wider animal health industry. The application of the tool showed that, for most diseases, a combination of both active and passive surveillance activities was appropriate for the surveillance programmes. For future use of the tool, prioritization on a per species basis with the relevant experts may be more advantageous than prioritization of surveillance programmes across species.

## Data Availability Statement

The original contributions presented in the study are included in the article/Supplementary Material, further inquiries can be directed to the corresponding author/s.

## Author Contributions

AC, DB, JM, AB, SM, and MH were involved in the design of the study. AC and DB lead the analysis and interpretation of the findings and drafting the manuscript. JM contributed to the preparation of the survey. All authors contributed to later versions of the manuscript and read and approved the final manuscript.

## Conflict of Interest

The authors declare that the research was conducted in the absence of any commercial or financial relationships that could be construed as a potential conflict of interest.
