# Supplementary material for: Development and Application of a Prioritization Tool for Animal Health Surveillance Activities in Ireland
Source: Front Vet Sci. 2020 Dec 23;7:596867. doi: 10.3389/fvets.2020.596867 (PMC7785526; doi:10.3389/fvets.2020.596867)
Supplement: Supplementary file 1 [file Data_Sheet_1.pdf]

Supplementary Material

A. Phase 1 Survey Questions

Question 1: Do you consent to participate in this study?

Under good operating standards, it should be recorded where respondents agreed to participate in a study. This question was placed first and if the participant ticked on the 'no' option, then no further questions could be answered in the survey.

Question 2: What is your current role?

The participants could select only one of five options. These included farm representative, state agency staff member, farm service provider, private vet or DAFM staff member. This question established the background from which the participants were coming from and if there were differences in their perspectives of the most important diseases.

Question 3: Twenty diseases/conditions, which are currently present (endemic) in Ireland, are listed randomly below. Please identify the 10 which you consider to be the most important in the Irish livestock industry and rank them in order of their importance. For example, if you consider Liver Fluke to be the most important click on 1, if you consider Besnoitia to be the second most important, click on 2. Please continue until you have selected the 10 most important (leaving the other 10 with no numbers assigned).

This question allowed each participant to identify the 10 most important endemic diseases and/or conditions and then to rank them in order of one to ten. Twenty diseases/conditions were listed randomly so to limit any bias when participants selected them. The survey monkey survey was set up so that any number between one and ten could only be used once, therefore no disease could have the same priority. It also ensured that the participants couldn't move to the next question until 10 diseases/conditions were chosen.

It has been acknowledged that disease importance is difficult to define. In a review by Brookes et al., (2015), it was stated that "diseases can have social, economic and environmental impacts, and most diseases cause a variety of impacts that span these broad categories. In addition, impacts vary in scale and are often intangible, and the perception of importance of each impact varies between

decision-makers". It is for this reason that the researcher did not put a specific definition on 'importance' in the survey. The researcher included a wide range of experts from different fields to identify the most important diseases. The variation in backgrounds would have allowed for impacts such as social, economic, environmental, and welfare been considered when experts choose their selection of the most important diseases.

Question 4: Excluding those listed in question 3, are there any other diseases/conditions currently present in Ireland that should be prioritised under DAFM surveillance programmes?

This question allowed the participant to identify any other endemic diseases that they felt should have been included in the top ten and that were not included in the list in the previous question. This allowed the researcher to consider any diseases that may have nominated by a number of the respondents, for the next survey (or in phase 2).

Question 5: Twenty diseases/conditions, which are currently absent from Ireland, are listed randomly below. Please identify the 10, which you consider to be the most important to the Irish livestock industry and rank them in order of their importance. For example, if you consider anthrax to be the most important, click on 1, if you consider rabies to the second most important click on 2. Please continue until you have selected the 10 most important (leaving the other 10 with no numbers assigned).

This question allowed the participant to identify the 10 most important exotic diseases and/or conditions and then to rank them in order of one to ten. Twenty diseases/conditions were listed randomly so to limit any bias when participants selected them. The survey monkey survey was set up so that any number between one and ten could only be used once, therefore no disease could have the same priority. It also ensured that the participants couldn't move to the next question until 10 diseases/conditions were chosen.

Question 6: Excluding those listed in question 5, are there any other diseases/conditions currently absent (exotic) that should be prioritised under DAFM surveillance programmes?

This question allowed the participant to identify any other exotic diseases that they felt should have been included in the top ten and that were not included in the list in the previous question. This allowed the researcher to consider any diseases that may have nominated by a number of the respondents, for the next step in the process.

B. Guidance Document accompanying the tool

Background information for the experts on the prioritisation tool:

Introduction

One of the recommendations from the Animal Health Surveillance Strategy for Ireland 2016-2021 is based on prioritisation and states that "DAFM should develop a prioritisation process for animal health surveillance activities and establish criteria by setting up a working group with stakeholders". In addition, with the pending introduction of the New Animal Law 2021 it is expected that DAFM will be required to prioritise animal health surveillance activities in the future.

A project is underway that comprised of two phases. The first phase involved a survey (using survey monkey) of stakeholders which represented DAFM staff members, state agency staff, farm service providers, farm organisation representatives and private vets. The participants were asked to rank in their opinion the ten most important endemic and the ten most important exotic diseases that face the agricultural industry in Ireland (excluding marine related diseases). Once these were established, the process of prioritising the surveillance activities for these diseases can take place which is phase two of the project. The accompanying tool developed in excel provides a simple methodology which experts may use to undertake the prioritization process.

The tool comprises two parts. Firstly, it will involve examining which surveillance objective is of a higher priority for each disease. Secondly it will examine which surveillance activities are best suited to meet the objectives of the surveillance for each disease within the constraints of the resources available for disease surveillance. This process can be completed by the chief veterinary officer and/or a number of experts working in animal health policy and can be repeated every few years to take account of progress in disease eradication and the potential emergence of new diseases. It should be noted that resources are allocated in a national context and not at farm level.

Part 1: Surveillance Objectives

There are four main objectives to animal health surveillance. These can be further categorised in two groups; diseases that are already present and diseases (endemic) that are currently absent (exotic) in a country (Table 1). Certain objectives are unique to each category. In the case of endemic disease, prevalence estimation and case finding are exclusive to diseases that are present (see table 1 below). For exotic diseases, early detection and proof of freedom are exclusive to diseases that are absent. It should be noted that there can also be crossover effects between the two objectives in each category. In practice the assessment is not a black and white scenario, however, for the analytical purposes of this study, they will be considered as four separate objectives.

Table 1: Most common surveillance objectives associated with livestock disease[1]

Disease	*Endemic*	*1. Prevalence estimation: Detection monitoring of endemic diseases for disease frequency estimation: describing the level of distribution of disease. This objective is focused on disease monitoring.*
		2. Case finding: Finding cases of a disease (at the animal or group/herd level). This objective is focused on disease eradication.
	Exotic	*3. Early detection: Detection of incursion of exotic, new (emerging) and re-emerging diseases. This objective is focused on the rapid detection of a disease not currently present.*
		4. Proof of freedom: Declaration of freedom from specified diseases and infections. This objective is to provide evidence that Ireland is free of a disease and is predominantly for trade purposes.

<u>*Surveillance Objectives Prioritisation*</u>

For endemic and exotic diseases, it is acknowledged that there are two surveillance objectives, and both have a role in the surveillance programme for each disease. However, for some diseases, one objective will be more important than the other, and for other diseases the two objectives will be relevant. Overtime the objectives may change for each disease. In Part 1 of the prioritisation tool, experts are asked to weight the current importance of these objectives out a total of 10, assigning a higher weight to the most important and then the remainder (out of 10) to the other objective.

[1] Table adapted from the RiskSur Best Practices for risk-based and cost-effective animal health surveillance in the European Union. Available : here

Part 2: Surveillance activities

There are two broad types of surveillance, active and passive.

Active Surveillance (DAFM led):

Active surveillance is the investigator-initiated collection of animal health related data through actions scheduled in advance using a defined protocol. These activities involve DAFM or a delegated party such as Animal health Ireland (AHI) leading programmes that actively test for a disease. Active surveillance activities include

- *Abattoir surveillance involving ante-mortem and post-mortem inspections of animals carried out by DAFM veterinary staff. These are undertaken for a variety of reasons including welfare and the detection of BSE and TB.*
- *Structured surveys which involve testing for a specific disease using a statistically significant representative sample of the population. This type of surveillance involves periodic surveys undertaken on farms (e.g. Brucellosis melitensis survey for sheep) or at slaughter plants (e.g. Brucellosis, EBL and BTV surveys for cattle, fluke surveys in lambs).*
- *Post importation checks of animals, animal products and animal by-products.*
- *Large scale case detection programmes involve the testing of the majority of animals in the population on a continual basis, for example case detection of TB and BVD, and BSE in over 48 month old fallen cattle. These fallen cattle are sampled for BSE by VIs from the local Regional Veterinary Offices (RVOs).*
- *Wildlife surveillance for the detection of TB in badgers and deer.*

Passive Surveillance:

Passive surveillance is the observer-initiated provision of animal health related data (e.g. voluntary notification of a suspect disease) or the use of existing data for surveillance. Passive surveillance activities include:

- *Regional Veterinary Offices have an important role in the carrying out the surveillance activities for a number of endemic and exotic diseases, where private veterinary surgeons and farmers may report suspect cases.*
- *Laboratory-based passive surveillance carried out by DAFM's six regional veterinary Laboratories (RVLs) and disease reporting by private veterinary practitioners to Regional Veterinary Offices for notifiable diseases such as BSE and exotic diseases, parasitology and the DAFM BVD eradication programme.*
- *Private Laboratories carry out many of the routine testing of samples, for example to identify the pathogen causing digestive problems in calves, parasitology and the DAFMs BVD eradication programme.*
- *Syndromic surveillance: Surveillance that uses health-related information (e.g. clinical signs or other data) that might precede (or may substitute for) formal diagnosis. This information may be used to indicate a sufficient probability of a change in the health of the population either to warrant further investigation or to enable a timely assessment of the impact of health threats which may require action. This type of surveillance is not usually focused on a particular hazard, so it can be used to detect a variety of diseases or pathogens- including new, emerging and re-emerging diseases. It is currently used more in public health than animal health, but it has a lot of potential in animal health given the large databases which are available.*

Surveillance Activities Prioritisation

The next step in the process is to identify and allocate resources to the activities that best meet the objectives of the surveillance programme. There is a lack of available data on the costings of the surveillance activities and a technique called proportional piling offers a different method of assigning a relative priority or value to different parameters. There is a limited amount of resources (financial and human) available for surveillance activities therefore one must allocate the resources to the activities within these limits. Experts are asked to allocate the resources across the activities by assigning 'chips' to the relevant activities for each disease. Using the proportional piling technique, 100 chips are available for each disease. The 100 chips should be considered as the total amount of resources (financial and human) currently available for each disease. Some activities will not be relevant for the surveillance of a disease and in these cases, no chips will be allocated.

The Centres for Disease Control and Prevention (CDC) guidelines have recommended that attributes including sensitivity, timeliness, data quality, representativeness, acceptability, flexibility, simplicity and usefulness are some of the criteria that can be used in the evaluation of surveillance systems (Drew et al., 2012). While it is noted this exercise is not an evaluation of the activities, these criteria may be useful to refer to, when experts are considering the activities and the allocation of resources to these activities during the prioritisation process.

C. Copy of Prioritisation Tool

Diseases/Conditions in Ireland

Part 1. It is acknowledged that both surveillance objectives have a role in the surveillance programme for each disease, however **which objective is currently the more important in the surveillance programme of each disease? Please assign a weight to each objective where the total of the two will equate to 10, but one will be a higher weight.**

Part 1	Surveillance Objectives		
Disease	Prevalence Estimation (for monitoring)	Case finding (for eradication)	Total must equal 10
Example: Disease A	3	7	10
Antimicrobial Resistance (AMR)			0
Bovine TB			0
Respiratory diseases			0
Johne's disease			0
Parasitism including liverfluke			0
Bovine Viral Diarrhoea (BVD)			0
Infectious Bovine Rhinotracheitis (IBR)			0
Bovine Spongiform Encephalopathy (BSE)			0
Neonatal enteritis			0
Equine herpesvirus infection			0

Part 2. The resources (financial and human) available for surveillance programmes are limited. Assuming a total of 100 chips for each disease, **how would you allocate the resources across the relevant activities to meet the objectives of the surveillance programme.** * Please refer to the accompanying document for further guidance on criteria to be considered.

Part 2	Surveillance Activities									
	Active Surveillance					Passive Surveillance				
Disease	Abattoir Surveillance	Large scale case detection programmes	Structured surveys	Importation checks	Wildlife surveillance	RVOs	DAFM Labs incl RVLs	Private labs	Syndromic Surveillance	Total must equal 100
Example Disease A	40	10	10	0	5	10	10	5	10	100
Antimicrobial Resistance (AMR)										0
Bovine TB										0
Respiratory diseases										0
Johne's disease										0
Parasitism including liverfluke										0
Bovine Viral Diarrhoea (BVD)										0
Infectious Bovine Rhinotracheitis (IBR)										0
Bovine Spongiform Encephalopathy (BSE)										0
Neonatal enteritis										0
Equine herpesvirus infection										0

Diseases/conditions not present in Ireland

Part 1	Surveillance Objectives		
Disease	Early Detection	Proof of Freedom	Total must equal 10
Example: Disease A	6	4	10
African Swine Fever (ASF)			0
Foot and Mouth Disease (FMD)			0
Bluetongue Virus (BTV)			0
Bovine Brucellosis			0
Rabies			0
Classical Swine Fever (CSF)			0
Avian Influenza			0
Equine Viral Arteritis (EVA)			0
Equine Infectious Anaemia (EIA)			0
Disease X (a previously unrecognised disease which could first emerge in Ireland)			0
			0

Part 1. It is acknowledged that both surveillance objectives have a role in the surveillance programme for each disease, however **which objective is currently the more important in the surveillance programme of each disease?** Please assign a weight to each objective where the total of the two will equate to 10, but one will be a higher weight.

Part 2. The resources (financial and human) available for surveillance programmes are limited. Assuming a total of 100 chips for each disease**, how would you allocate the resources across the relevant activities to meet the objectives of the surveillance programme.*** Please refer to the accompanying document for further guidance on criteria to be considered.

Part 2	Surveillance Activities									
	Active Surveillance					Passive Surveillance				
Disease	Abattoir Surveillance	Large scale case detection programmes	Structured surveys	Importation checks	Wildlife surveillance	RVOs	DAFM Labs incl RVLs	Private Labs	Syndromic Surveillance	Total must equal 100
Example Disease A	50	0	10	0	5	10	10	5	10	100
African Swine Fever (ASF)										0
Foot and Mouth Disease (FMD)										0
Bluetongue Virus (BTV)										0
Bovine Brucellosis										0
Rabies										0
Classical Swine Fever (CSF)										0
Avian Influenza										0
Equine Viral Arteritis (EVA)										0
Equine Infectious Anaemia (EIA)										0
Disease X (a previously unrecognised disease which could first emerge in Ireland)										0

D. Detailed results showing allocation of resources to individual surveillance activities

Table D1: Allocation of resources to surveillance activities for endemic diseases/conditions

*highlighted cell shows the activities that was allocated the most resources for each disease.

	Active Surveillance						Passive Surveillance			
Disease	Abattoir Surveillance	Large scale case detection programmes	Structured surveys	Importation checks	Wildlife surveillance		RVOs	DAFM laboratories incl. RVLs	Private laboratories	Syndromic Surveillance
African Swine Fever (ASF)	**10**	**4**	**5**	**29**	**3**		**4**	**22**	**4**	**22**
Avian Influenza	**4**	**4**	**20**	**6**	**22**		**8**	**16**	**2**	**18**
Bluetongue Virus (BTV)	**10**	**2**	**25**	**28**	**5**		**7**	**10**	**3**	**10**
Bovine brucellosis	**18**	**4**	**18**	**14**	**0**		**6**	**15**	**3**	**20**
Classical Swine Fever (CSF)	**16**	**2**	**19**	**22**	**0**		**6**	**14**	**5**	**15**
Disease X	**6**	**2**	**7**	**5**	**10**		**8**	**19**	**12**	**32**
Equine Infectious Anaemia (EIA)	**1**	**6**	**11**	**19**	**4**		**4**	**25**	**19**	**11**
Equine Viral Arteritis (EVA)	**1**	**5**	**12**	**19**	**0**		**6**	**31**	**21**	**6**
Foot and Mouth Disease (FMD)	**16**	**3**	**4**	**18**	**1**		**14**	**17**	**3**	**25**
Rabies	**1**	**1**	**5**	**33**	**9**		**6**	**14**	**2**	**28**

Table D2: Allocation of resources to surveillance activities for exotic diseases/conditions

*highlighted cell shows the activities that was allocated the most resources for each disease.

	Active Surveillance						Passive Surveillance			
Disease/Condition	Abattoir Surveillance	Large scale case detection programmes	Structured surveys	Importation checks	Wildlife surveillance		RVOs	DAFM laboratories incl. RVLs	Private laboratories	Syndromic Surveillance
Antimicrobial Resistance (AMR)	11	7	28	0	5		2	24	18	7
Bovine Spongiform Encephalopathy (BSE)	38	10	6	1	1		12	16	5	14
Bovine TB	16	46	0	2	11		10	11	1	3
Bovine Viral Diarrhoea (BVD)	4	57	8	5	1		3	10	9	5
Equine herpesvirus infection	1	6	18	10	0		4	14	37	10
Infectious Bovine Rhinotracheitis (IBR)	7	40	15	4	1		3	15	12	5
Johne's disease	5	41	15	5	1		5	12	9	9
Neonatal enteritis	0	4	13	1	1		4	39	23	16
Parasitism including liverfluke	48	5	8	0	1		2	17	8	10
Respiratory diseases	17	9	13	2	2		4	25	13	18
